# A Distinct Expression Pattern in Mammalian Testes Indicates a Conserved Role for NANOG in Spermatogenesis

**DOI:** 10.1371/journal.pone.0010987

**Published:** 2010-06-07

**Authors:** Ewart W. Kuijk, Jeffrey de Gier, Susana M. Chuva de Sousa Lopes, Ian Chambers, Ans M. M. van Pelt, Ben Colenbrander, Bernard A. J. Roelen

**Affiliations:** 1 Department of Farm Animal Health, Faculty of Veterinary Medicine, Utrecht University, Utrecht, The Netherlands; 2 Department of Reproductive Medicine and Gynecology, University Medical Center Utrecht, Utrecht, The Netherlands; 3 Hubrecht Institute-KNAW and University Medical Center Utrecht, Utrecht, The Netherlands; 4 Department of Clinical Sciences of Companion Animals, Faculty of Veterinary Medicine, Utrecht University, Utrecht, The Netherlands; 5 Department of Anatomy and Embryology, Leiden University Medical Center, Leiden University, Leiden, The Netherlands; 6 Medical Research Council Centre for Regenerative Medicine, Institute for Stem Cell Research, School of Biological Sciences, University of Edinburgh, Edinburgh, United Kingdom; 7 Center for Reproductive Medicine, Department of Obstetrics and Gynecology, Academic Medical Center, University of Amsterdam, Amsterdam, The Netherlands; City of Hope National Medical Center, United States of America

## Abstract

**Background:**

NANOG is a key player in pluripotency and its expression is restricted to pluripotent cells of the inner cell mass, the epiblast and to primordial germ cells. Spermatogenesis is closely associated with pluripotency, because through this process highly specialized sperm cells are produced that contribute to the formation of totipotent zygotes. Nevertheless, it is unknown if NANOG plays a role in this process.

**Methodology/Principal Findings:**

In the current study, NANOG expression was examined in testes of various mammals, including mouse and human. *Nanog* mRNA and NANOG protein were detected by RT-PCR, immunohistochemistry, and western blotting. Furthermore, eGFP expression was detected in the testis of a transgenic *Nanog eGFP*-reporter mouse. Surprisingly, although NANOG expression has previously been associated with undifferentiated cells with stem cell potential, expression in the testis was observed in pachytene spermatocytes and in the first steps of haploid germ cell maturation (spermiogenesis). Weak expression in type A spermatogonia was also observed.

**Conclusions:**

The findings of the current study strongly suggest a conserved role for NANOG in meiotic and post-meiotic stages of male germ cell development.

## Introduction

NANOG, named after a mythical Celtic land of the ever young, is a homeobox transcription factor and a key player in pluripotency; i.e. the ability of a cell to differentiate into any fetal or adult cell type [1], [2]. NANOG is expressed in pluripotent cell lines, including embryonic stem (ES) cells and embryonic germ (EG) cells and loss of NANOG results in a tendency of ES cells to differentiate [1]–[3]. On the other hand, mouse ES cells that constitutively express *Nanog* are less sensitive to differentiation signals and do not require LIF to maintain an ES cell identity [2]. Fusion of somatic cells with ES cells that express elevated levels of NANOG facilitates reprogramming of the restricted somatic genome to a pluripotent state [4].

*In vivo* expression of NANOG is restricted to cells of the inner cell mass (ICM), the pluripotent epiblast, and primordial germ cells (PGCs), all of which are sources for pluripotent cell lines [1], [2], [5]. In mouse development, NANOG becomes detectable in migrating PGCs of E7.75–E8.0 embryos. In gonadal germ cells of E13.5 and E14.5 female embryos, NANOG is undetectable in cells positive for the synaptonemal complex-specific protein SCP3. In germ cells of E14.5–E16.5 male embryos, NANOG expression is lost during the mitotic arrest [5].

The ICM cells of *Nanog* ^−/−^ embryos fails to acquire pluripotency and does not develop into pluripotent epiblast cells [1], [6]. When put into culture, explanted ICM cells differentiate entirely into extraembryonic endoderm lineages [1]. Remarkably, however, *Nanog* ^−/−^ ICM cells can participate in chimaeric embryonic development and contribute to all three embryonic germ layers. This demonstrates that in pluripotent cells NANOG is dispensable for the potential to differentiate into all somatic tissues. However, in these chimaeras, the *Nanog* ^−/−^ cells that became committed to the primordial germ cell lineage did not survive beyond E11.5, indicating that *Nanog* is indispensable for the development of postmigratory germ cells [3].

Somatic cells that are induced to pluripotency by the introduction of exogenous reprogramming factors do not require exogenous NANOG for this process [7]. However, *Nanog* ^−/−^ neuronal stem cells can not be reprogrammed to pluripotency, unless they carry a transgene that expresses NANOG [6]. It seems that *Nanog* is dispensable for the initial dedifferentiation to occur, but essential for the final stage of reprogramming to allow pluripotency [6].

Male germ cells appear to be an important source of cells that are directly linked to pluripotency. Teratocarcinomas, for example, are malignant germ cell tumors that are composed of pluripotent cells and differentiated progeny of numerous cell types [8]. Moreover, a number of recent reports describe the derivation of pluripotent cells from neonatal and adult mouse [9]–[13] and human [14]–[16] testes. However, the immediate link between testis and pluripotency is evidently spermatogenesis: the production of sperm cells that can fertilize oocytes and thereby contribute to the formation of completely new organisms.

Despite the evidence for the expression of *Nanog* in the testes of several species [17]–[20] and obvious link between testis function and pluripotency, little is known about the involvement of NANOG in the process of spermatogenesis. In the current study we describe NANOG expression in the testis and determine its expression pattern in mammalian species ranging from mouse, dog, and pig, to human. The dynamics of NANOG expression throughout spermatogenesis was similar between species suggesting a conserved role for NANOG in male meiosis and spermiogenesis.

## Results

**Figure 1:**
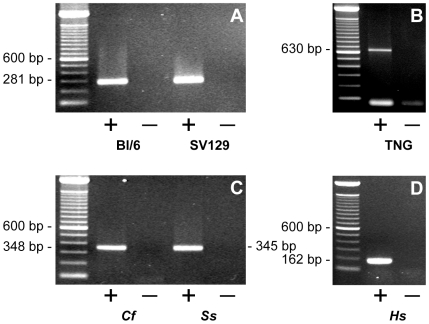
Expression of *Nanog* in testis of various mammalian species as determined by RT-PCR. (A) Expression of *Nanog* in mouse testis of the Black 6 strain (BL/6) and the SV129 strain (SV129) (B) Expression of *eGFP* in testis of a TNG mouse; (C) Expression of *NANOG* in dog and pig testes; (D) Expression of *NANOG* in human testis; in all RT-PCR experiments, amplicons were of the expected sizes (See also Table 2) and products were identified by sequence analysis. Abbreviations: TNG =  testis cDNA from *Nanog* eGFP reporter mouse; *Cf* =  *Canis familiaris*, *Ss* =  *Sus scrofa*, *Hs* =  *Homo sapiens*; +  = cDNA synthesis reaction performed with reverse transcriptase, −  =  minus reverse transcriptase control.

We detected the presence of *Nanog* mRNA in mouse testes of two different strains by reverse transcriptase-polymerase chain reaction (RT-PCR) (Fig. 1A). Transcription from the locus of *Nanog* in mouse testes was further confirmed by the detection of testicular eGFP transcripts in transgenic *Nanog eGFP*-reporter mice (TNG) [3] (Fig. 1B). Further investigation in other species revealed *NANOG* expression in dog, pig, and human testes (Fig. 1C, D). Importantly, rigorous negative controls demonstrated that PCR products were not derived from genomic DNA.

**Figure 2:**
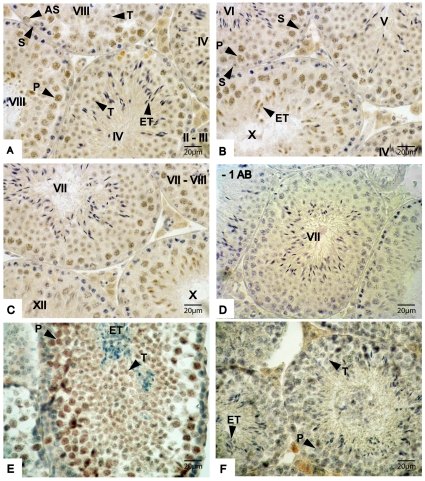
Expression of NANOG and eGFP in mouse testes sections as detected by immunohistochemistry. (A–C) Expression of NANOG in mouse testis at different stages of spermatogenesis; Roman figures in seminiferous tubules represent the stage of the epithelial cycle; (D) Negative control section from which the primary antibody was omitted in the staining procedure; (E) Expression of eGFP in testis sections of a *Nanog* eGFP reporter; (F) eGFP staining on testis sections of a non-transgenic mouse, which served as a negative control for the staining in figures E. Abbreviations: AS =  type A spermatogonia, P =  Pachytene spermatocyte, T = Spermatid, ET =  elongating spermatids, S =  Sertoli cell, * =  non-specific binding to interstitial cells. Sections displayed in panels A–D were fixed in Bouins fixative; sections in panels E and F were fixed in formalin. Formalin fixation interfered with immunostaining of the seminiferous tubules.

Localization of NANOG protein expression was performed on mouse testis sections using immunohistochemistry and expression was detected in the nucleus of pachytene spermatocytes and round spermatids (Fig. 2A–C). Weak staining for NANOG was also detected in type A spermatogonia in all stages of the epithelial cycle (e.g. Fig. 2A), but NANOG was not detected in intermediate spermatogonia (data not shown). Control sections in which the primary antibody solution was replaced by blocking solution did not show any signal in the seminiferous tubules (Fig. 2D). Next, we examined eGFP expression in testes sections of TNG mice. Although fluorescent cells were not detected by microscopy or FACS (data not shown) expression of eGFP was detected by immunohistochemistry. The cell types expressing eGFP in the testis of *Nanog* reporter mice corresponded to the cell types expressing NANOG (Fig. 2E). As expected, eGFP staining was not observed in testis sections of a non-transgenic mouse.

**Figure 3:**
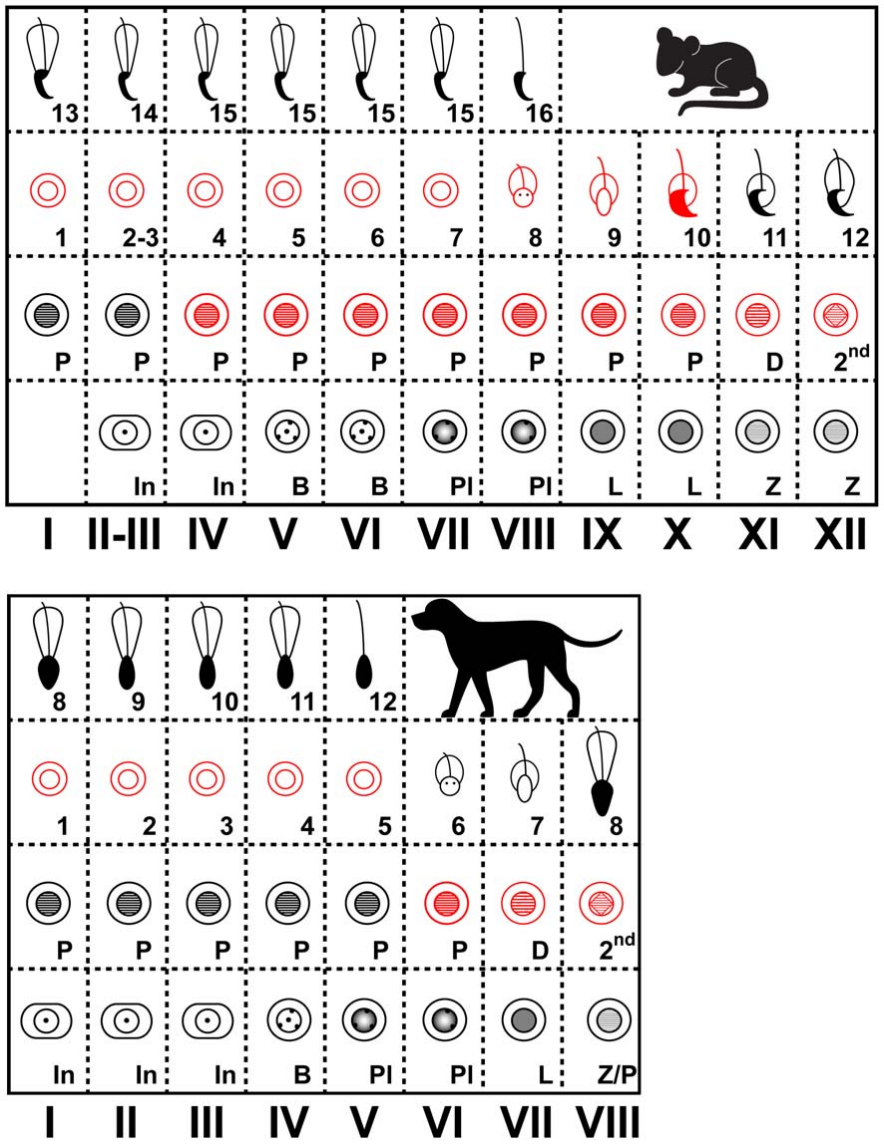
Models for the dynamic expression of NANOG in the epithelial cycle of murine (top) and canine (bottom) spermatogenesis. Each column represents the combination of different cell types that are present in seminiferous tubules at that specific stage. Roman figures =  stage of the epithelial cycle, In =  intermediate spermatogonia, B =  type B spermatogonia, Pl =  pre-leptotene stage, L =  leptotene stage, Z =  zygotene stage, P =  pachytene stage, D =  diplotene stage, 2^nd^ =  generation of secondary spermatocytes, 1–16 =  steps in spermiogenesis. Cell types that express NANOG are outlined in red and cell types that do not express NANOG have black and grey symbols.

Categorization of the cell types that express NANOG in the spermatogenic stages of the seminiferous epithelium [21] revealed a distinctive temporal expression pattern. The expression dynamics of NANOG in mouse testes at the various epithelial stages of spermatogenesis are summarized in Figure 3A. NANOG was detected in nuclei of pachytene spermatocytes from epithelial stage IV onwards. Expression was maintained throughout meiosis and NANOG was detected until spermiogenic step 10. Expression was progressively lost in the course of elongation of spermatids.

**Figure 4:**
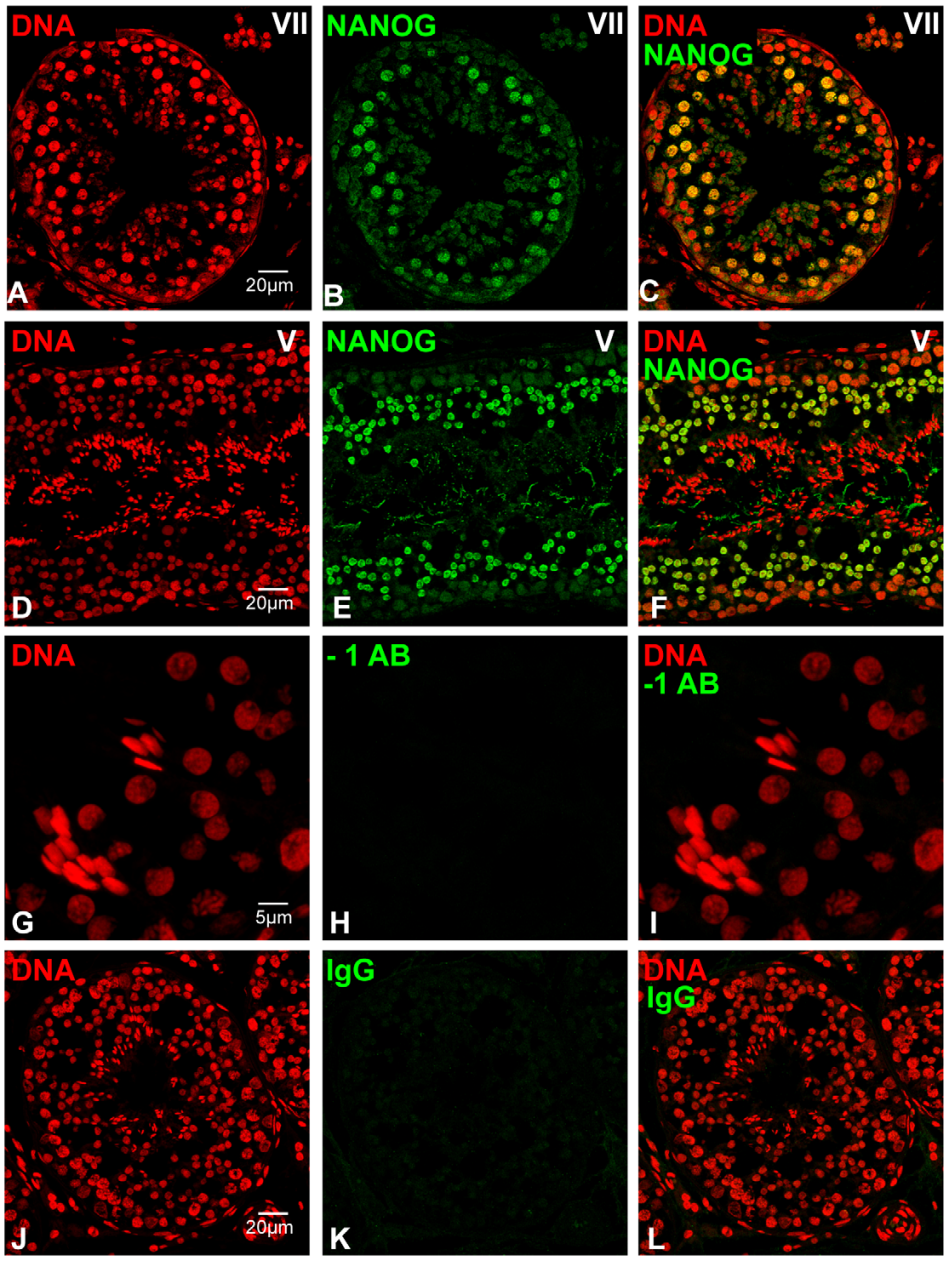
Expression of NANOG in dog testis as determined by immunofluorescence. (A–F) NANOG expression in differentiating male germ cells. Roman figures in panels represent the stage of the canine epithelial cycle (G–I) high magnification image of section in which the primary antibody was replaced by blocking solution, to control for non-specific binding of the secondary antibody; (J–L) image of testis section that was incubated with a rabbit IgG isotype control, to control for non-specific binding of the primary antibody.

We next analyzed the localization of NANOG expression in dog testis. The expression pattern resembled the patterns observed in the mouse testes and NANOG was mainly detected in maturing gametes (Fig. 4). NANOG was specifically expressed in pachytene spermatocytes and round spermatids of dog testis with a dynamic temporal expression pattern in the spermatogenic stages of seminiferous tubules. At canine epithelial stage V, just before spermatids initiate elongation [21], NANOG was expressed in pachytene spermatocytes, but not in elongating spermatids (Fig. 4A–C). The meiotic divisions in dogs occur at epithelial stage VIII and metaphase configurations and newly formed round spermatids were positive for NANOG at this stage. Spermatids remained positive for NANOG up until stage V (Fig. 4D–F). The expression dynamics of NANOG in dog testis at the various epithelial stages of spermatogenesis are summarized in Figure 3B, which illustrates that the observed expression pattern for NANOG in dog testis corresponds well to that observed in the mouse.

**Figure 5:**
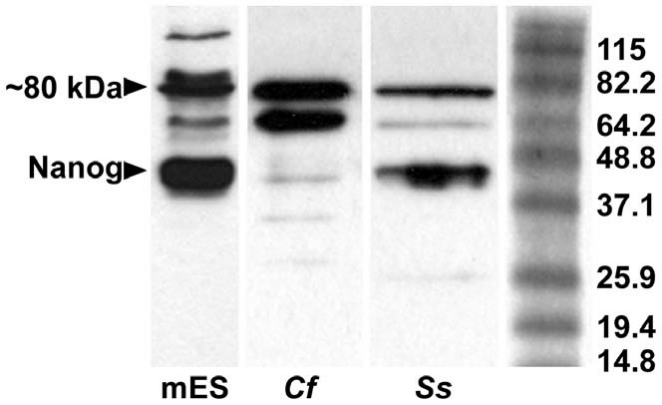
Immunoblot analysis. Composite image of immunoblot results for NANOG on lysates of mouse ES cells and dog and pig testes. On the right is a Benchmark protein ladder (Invitrogen) and the indicated molecular weights for each band. Abbreviations: mES =  mouse embryonic stem cells, *Cf* =  *Canis familiaris*, *Ss* =  *Sus scrofa*.

The presence of NANOG protein in dog testis was confirmed by immunoblot analysis (Fig. 5). A distinctive band of approximately 40 kDa, which corresponds to the molecular weight of NANOG, was observed in lysates from mES cells and dog testis. Additional bands in both mES cell and dog testis lysates were observed at approximately 80 kDa.

**Figure 6:**
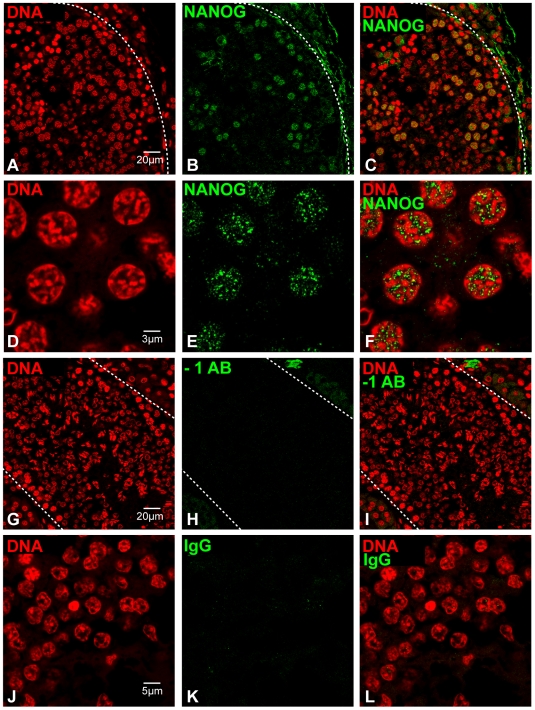
Expression of NANOG in pig testis as determined by immunofluorescence. (A–C) NANOG expression in differentiating male germ cells; (D–F) high magnification image of NANOG expression in pachytene spermatocytes; (G–I) image of section in which the primary antibody was replaced by blocking solution, to control for non-specific binding of the secondary antibody; (J–K) representative high magnification image of testis section that was incubated with a rabbit IgG isotype control, to control for non-specific binding of the primary antibody. Dashed lines in panels A–C and G–I mark the boundaries of the tubules.

**Figure 7:**
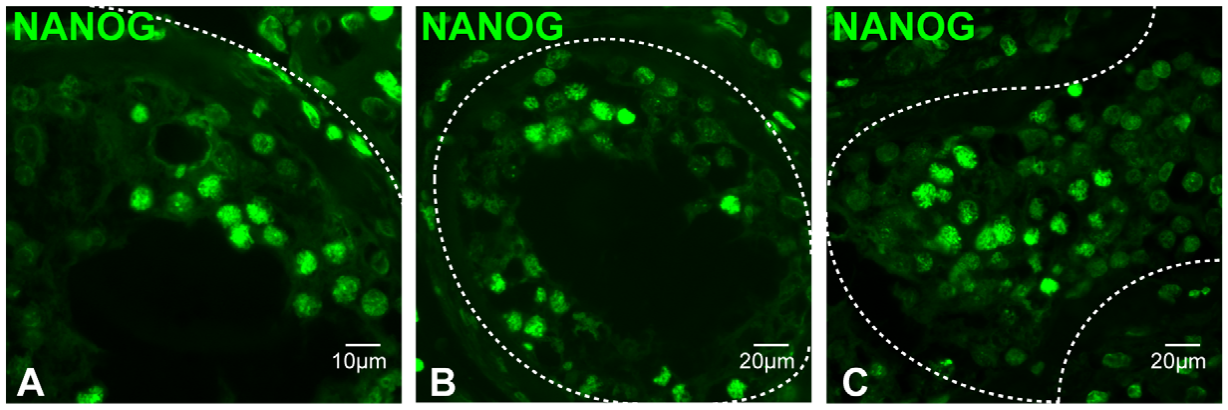
Expression of NANOG in human testis as determined by immunofluorescence in paraffin-embedded sections. (A–C) NANOG expression in differentiating male germ cells; dashed lines mark the boundaries of tubules.

NANOG expression was also detected in maturing gametes in adult porcine testis, as determined by immunofluorescence on paraffin-embedded sections (Fig. 6). Expression was detected in pachytene spermatocytes, as determined by the morphology of their DNA (Fig. 6A–F). Protein expression of NANOG in porcine testis was further confirmed by immunoblot analysis (Fig. 5). Additional bands in both mES cell and pig testis lysates were observed at approximately 80 kDa. NANOG protein expression was also detected in human testes as determined by immunofluorescence (Fig. 7). As in the other species examined, NANOG was localized to differentiating male germ cells.

## Discussion

NANOG is an essential factor for establishing pluripotency [6]. Recent studies have demonstrated that, in addition to cells of the ICM, the epiblast, primordial germ cells, and cultured male germ cells from mouse and human testes can give rise to pluripotent stem cells [9]–[16]. In the current study, we examined the expression of NANOG in male germ cells, since NANOG is expressed in other cell types from which pluripotent cell lines have been established [1], [2], [5], [18].

*Nanog* transcripts and protein were detected in testes of mouse, dog, pig, and human. Furthermore, *eGFP* mRNA and protein were detected in the testis of *Nanog* reporter mice, which is strong evidence that the *Nanog* gene is transcribed in mouse testis.

Multiple antibodies of different origins showed specific and comparable staining for NANOG in different species. Moreover, the observed expression in type A spermatogonia, spermatocytes, and round spermatids corresponds well to that described in previous studies of human [19] and porcine testes [20]. Importantly, the antibody that was used by Ezeh et al. (2005) was raised against a different epitope compared to the various antibodies that were used in our study. Consequently, it is unlikely that the signals described here originate from non-specific binding. In some previous studies, *Nanog* expression was not detected in mouse testis, even though expression was observed in cells of the ICM, the epiblast, and in primordial germ cells [1], [5]. Another study has detected NANOG in human fetal gonocytes, testicular carcinoma *in situ* and germ cell tumours, but not in normal adult testis [22]. Similarly, NANOG expression has also been detected in fetal gonocytes of the marmoset, but not in adult testis [23]. This probably indicates that the expression levels described here are relatively weak in differentiating male germ cells. Indicative of this low expression is the lack of detection of fluorescent cells by microscopy or FACS (data not shown) in TNG mouse testis. Nevertheless, eGFP could clearly detected by immunohistochemistry in TNG mouse testis, but not in the testis of non-transgenic mice. The sensitivity of the various approaches in the current study could have been decisive for the successful detection of NANOG in testes.

In transgenic mice that can express a short hairpin RNA against *Nanog* after tamoxifen exposure, conditional knockdown of *Nanog* in migratory PGCs induces apoptotic cell death and results in a reduction of the number of PGCs in E12.5 embryos [24]. The authors of this study also describe that embryonic Cre-recombinase mediated down regulation of *Nanog* resulted in tubules with disrupted spermatogenesis in the adult testes, which contained scattered TRA98-positive spermatogonia in the otherwise empty tubules. The authors interpreted this phenotype as developmental retardation of the seminiferous tubule probably caused by the embryonic loss of *Nanog*-negative germ cells [24]. However, the observed phenotype could also indicate a role for *Nanog* in spermatogenesis. The lack of meiotic and post meiotic stages correlates with our data on the onset of NANOG expression in pachytene spermatocytes.

In the course of germ cell development, male germ cells undergo critical epigenetic changes intended for successful progression through meiosis, meiotic sex chromosome inactivation, post-meiotic sex-chromosome repression, and chromatin remodeling [25]. The fundamental role of histone and DNA methyltransferases and other epigenetic modifiers in male meiosis is exemplified by various knockout studies that result in arrest and/or apoptosis of early to late pachytene spermatocytes [25], [26]. NANOG could be involved in these epigenetic events in spermatogenesis. For example, NANOG is known to interact with repression complexes that contain active histone deacetylases [27], [28]. Inhibition of histone deacetylase-1 results in male infertility through apoptosis in spermatogonia and spermatocytes [29], [30].

In conclusion, in the present study we demonstrated that *Nanog* is expressed in mammalian differentiating male germ cells. In mouse testis, weak expression in type A spermatogonia was also observed. The reported meiotic and post-meiotic association of NANOG in male germ line cells suggests a role for NANOG as epigenetic modifier in spermatogenic reprogramming.

## Materials and Methods

### Ethics statement

After institutional approval, mouse testes were obtained from fertile males that were housed at the Utrecht University Central Animal Facilities and the MRC Center Development in Stem Cell Biology, Institute for Stem Cell Research, School of Biological Sciences, University of Edinburgh. Because we made use of post-mortem surplus material from animals that were used for other experiments, ethical approval to use this material was not necessary. Surplus canine testicles were obtained from healthy client-owned dogs that underwent routine elective gonadectomy, performed by surgeons of the Department of Clinical Sciences of Companion Animals, Faculty of Veterinary Medicine, Utrecht, The Netherlands. Adult pig testes were collected at a local slaughterhouse. Anonym human testis samples for RNA isolation were obtained at the Center for Reproductive Medicine, Academic Medical Center, Amsterdam, from 2 patients undergoing bilateral castration as part of prostate cancer treatment. Patients were asked to donate this spare material for research and gave oral consent. Written consent was not considered necessary because the material would otherwise be discarded and because the consent of the patient was documented in their medical files. Ethics committee approval was not obtained, because according to Dutch law this is not necessary when anonym tissue samples are used. None of these men had previously received chemotherapy or radiotherapy, and the morphology of the testes showed normal spermatogenesis in all cases. Anonymized, sections embedded in paraffin of post-mortem human testes were obtained from the Biobank at the Department of Pathology of the University Medical Center in Utrecht. Approval to use this material for the current study was obtained from the scientific committee of the Department of Pathology of the University Medical Center in Utrecht (PA code RP 2007-34). There were no indications that this material was derived from infertile men or men suffering from testicular diseases as determined by testis morphology.

### RNA extraction, reverse transcription, and PCR

From human testes, mRNA was extracted using the MagNA Pure LC (Roche, Basel, Switzerland) according to the manufacturer's protocol. For cDNA synthesis 0.03 µg of mRNA of human testis tissue was used in a reverse transcriptase reaction with random primers and M-MLV reverse transcriptase (Invitrogen, Groningen, The Netherlands). For mouse, dog, and pig, total RNA was isolated from 50–100 mg testis sections with Trizol (Invitrogen), according to the manufacturer's protocol with an additional purification step immediately following phase separation. In this extra step, one volume of phenol:chloroform:isoamyl alcohol (Fluka, Sigma-Aldrich, Zwijndrecht, The Netherlands) was added to the aqueous phase and samples were mixed, incubated at room temperature (RT) for 5 min, and centrifuged. Subsequently, samples were incubated at RT for an additional 5 min, after which the aqueous phase was transferred to a new tube and put on ice. From here, the Trizol RNA isolation protocol was resumed, from the RNA precipitation step onwards. The presence of *Nanog* pseudogenes requires reliable negative controls in RT-PCR experiments [31]. Therefore, RNA was treated with 2 µl DNAse (2,75 Kunitz units/µl; Qiagen, Venlo, The Netherlands) for 20min at 37°C, after which incubation at 65°C for 10 min was performed to inactivate the enzyme. Next, RNA was reverse-transcribed to first strand cDNA with Superscript III (Invitrogen) according to the company's instructions. Random primers were used and for each sample an equivalent mixture was prepared, from which Superscript III was omitted, to control for genomic DNA contamination. Reverse transcribed cDNA samples were stored at −20°C before they were used in a polymerase chain reaction. Species-specific primers for *Nanog* were designed with Beacon Designer 4 (PREMIER Biosoft International, Palo Alto, CA, USA; (Table 1).

**Table 1:** Primers used for RT-PCR and sequence analysis.

Gene	Sequence origin	Primers	Ta (°C)	Amplicon size (bp)	Application
Mouse *Nanog*	NM_028016	F: 5′-AGATGCGGACTGTGTTCTC-3′R: 5′-TGCGTTCACCAGATAGCC-3′	58	281	PCR/sequence
Canine *NANOG*	XM_543828	F: 5′-CCGTCTCTCCTCTTCCTTC-3′R: 5′-CACTGTTGCTCTCCTTTGG-3′	54,3	348	PCR/sequence
Porcine *NANOG*	NM_001129971	F: 5′-CTCTCCTCTTCCTTCCTC-3′R: 5′-ATCACACTGTTGCTATTCC-3′	58	345	PCR/sequence
Human *NANOG*	NM_024865	F: 5′-CCTCCAGCAGATGCAAGAACTC-3′R: 5′-GTAAAGGCTGGGGTAGGTAGGTG-3′	58	172	PCR/sequence
eGFP	EU541500	F1: 5′-CTGGTCGAGCTGGACGGCGACG-3′R1: 5′-CACGAACTCCAGCAGGACCATG-3′	59	630	PCR/sequence

F =  forward primer, R =  reverse primer. Ta =  annealing temperature used in PCR reaction.

Complementary DNA amplification was performed with HotStarTaq (Qiagen) according to the manufacturer's protocol. The reaction mixture contained 2 nM MgCl2 and 0.5 µM of each primer. To 24 µl of mixture, 1 µl of sample was added, after which the PCR was carried out in a MyCycler (Bio-Rad). Each PCR started with a 15-min dwell at 94°C, followed by 40 cycles with 3 steps/cycle; 30 sec melting of double strands at 94°C, 30 sec annealing at the primer specific annealing temperature (Table 1), and finally 30 sec elongation at 72°C. For each sample, RT negative controls were used in all reactions to prevent contamination with genomic DNA. The amplicons (10 µl) were electrophoresed in 1% agarose gels and sequence analysis was used to confirm the amplification of *Nanog* transcripts in all species.

### Immunoblot analysis

Total protein was extracted with lysis buffer containing 25 mM 2-(N-morpholino) ethanesulfonic acid monohydrate (Sigma-Aldritch), 150 mM NaCl, 1 mM EGTA, protease inhibitors (Roche), and 1% Triton X-100. The amount of protein was determined by a DC protein assay (Bio-Rad) and 5–20 µg of total protein, was boiled for 5 min in Laemmli buffer (Bio-Rad) with 0.5 M β-mercaptoethanol and separated by electrophoresis on a 12% Tris–HCl PAGE gel. To determine protein sizes, a benchmark pre-stained protein ladder was run in each gel. After electrophoresis, proteins were transferred to a Trans-Blot nitrocellulose transfer membrane (Bio-Rad). Subsequently, membranes were blocked overnight at 4°C with 5% Blotting Grade Blocker non-fat dry milk (Bio-Rad), in PBST and incubated with 1 µg/ml rabbit polyclonal anti-mouse NANOG (Chemicon) in 5% non-fat dry milk for 1 hr at room temperature. Membranes were washed in PBST, and subsequently incubated for 1 hr at RT in a horseradish peroxidase-conjugated goat anti-rabbit IgG (Pierce, Rockford, USA) secondary antibody solution diluted 1∶5000 in 5% non-fat dry milk. Specific binding of the antibodies was visualized using SuperSignal West Dura Extended Duration Substrate (Pierce, Rockford, IL) after which the blots were exposed to film (Kodak). The primary antibody is known to bind non-specifically to a 55 kDa protein and therefore, bands detected at that height were considered non-specific.

### Immunohistochemistry, Immunofluorescence, and Confocal Laser Scanning Microscopy

After collection, testes were fixed overnight (o/n) in 4% PFA or Bouins fixative and embedded in paraffin using standard procedures the following day. Sections were cut at 5–7 µm and mounted on superfrost plus slides (Menzel, Braunschweig, Germany), which were dried o/n at 37°C and stored at 4°C until further use. For IHC with the NANOG antibody (Table 2) on mouse sections, endogenous peroxidases were blocked with 1.5% H_2_O_2_ (Merck) in 40 mM citric acid and 120 mM Na_2_HPO_4_ for 15 min at RT. After antigen retrieval with 10 mM EDTA buffer (20 min, pH 9), non-specific binding sites were blocked with 1% bovine serum albumin in PBS for 30 min at RT. After blocking, sections were incubated with rabbit anti-nanog (Bethyl, Montgomery, USA) in blocking solution overnight at 4°C. Slides were incubated with a Powervision poly-HRP-anti-rabbit conjugated secondary antibody (ImmunoVision Technologies) for 1 hr at RT. Subsequently, sections were incubated in Fast 3,3′-diaminobenzedine (Sigma-Aldrich) and counterstained with heamatoxylin. Slides were mounted with Pertex (Klinipath, Duiven, The Netherlands).

**Table 2:** Antibodies used for immuno applications.

Immunogen	Source	Concentration	Description	Application
AA1-50 mouse NANOG	A300-398A (Bethyl)	10 µg/ml	Rabbit polyclonal	IHC mouse
Full length mouse NANOG fusion protein	AB21603 (Abcam)	1–2 µg/ml	Rabbit polyclonal	IF Dog, human
Synthetic peptide mouse NANOG	AB5731 (Chemicon)	1 µg/ml	Rabbit polyclonal	IB, IF pig
Human NANOG	14-5768 (eBioscience)	1–2 µg/ml	Mouse monoclonal	IF human
GFP (B-2)	SC9996 (Santa Cruz)	1∶500	Mouse monoclonal	IHC TNG mouse
Rabbit IgG	31460 (Pierce)	0.16 µg/ml	Goat polyclonal peroxidase conjugated	IB

AA =  Amino Acids, IHC =  immunohistochemistry, IF =  immunofluorescence, IB = immunoblotting.

For immunohistochemistry with the GFP antibody (Table 2), PFA fixed mouse sections were deparaffinized after which endogenous peroxidases were blocked with methanol/0.3% H_2_O_2_. Next, antigen retrieval was performed by boiling the slides for 10 min in EDTA buffer (1 mM, pH 9). Endogenous biotin was blocked by incubation of the sections with avidin and biotin, respectively. Subsequently, non-specific binding was blocked with 10% normal goat serum in 0.05% Tween in PBS (PBST) for 30 min at RT after which the blocking solution was replaced by primary antibody solution diluted in 2% normal goat serum (NGS) in PBST (60 min at RT). Sections were then incubated in biotinylated goat anti-mouse IgGs (1∶100; Dako, Heverlee, Belgium) in 2% NGS in PBST for 30 min at RT. After 20 min incubation with horseradish peroxidase (HRP)-conjugated streptavidin (DAKO) in 2% NGS in PBST, slides were incubated in AEC solution (Sigma-Aldrich) and counter stained with hemaetoxylin.

For immunofluorescence, slides were deparaffinized in xylene and subjected to antigen retrieval by boiling the slides for 10 min in citrate buffer (pH 2 or pH 6). For canine testis sections, additional antigen retrieval was performed by putting the slides in methanol for 10 sec. Following antigen retrieval, slides were permeabilized in TBS containing 0.05% Tween (TBST) and Triton X-100 (0.1%). Permeabilized slides were blocked for 1 hr in TBST containing 0.5% BSA (Sigma-Aldrich) followed by o/n incubation with the primary antibody (Table 2) in blocking solution at 4°C. The following day, slides were incubated in the secondary antibody in blocking solution for 1 h at 4°C, after which the slides were counterstained with TOPRO-3 (Invitrogen) and mounted in Vectashield. DNA could not be visualized after antigen retrieval at pH 2.

Fluorescent images were analyzed at the Center for Cell Imaging at the Faculty of Veterinary Medicine in Utrecht. Fluorescent signals were visualized using a Confocal Laser Scanning Microscope (Bio-Rad) and on an epifluorescence microscope from which pictures were captured with a CCD camera. Within each session, identical settings were used to image NANOG stains and negative controls. For post-capture analysis, only brightness and contrast were used to enhance signals and negative control images were treated in an equal manner.
